# Supplementary figures and images for: Comparing the growth and yield performance of six different varieties of frafra potato (Solenostemon rotundifluis Poir) grown under rain-fed conditions in the Guinea Savanna ecological zone of Ghana
Source: PLoS One. 2022 Nov 16;17(11):e0276566. doi: 10.1371/journal.pone.0276566 (PMC9668201; doi:10.1371/journal.pone.0276566)

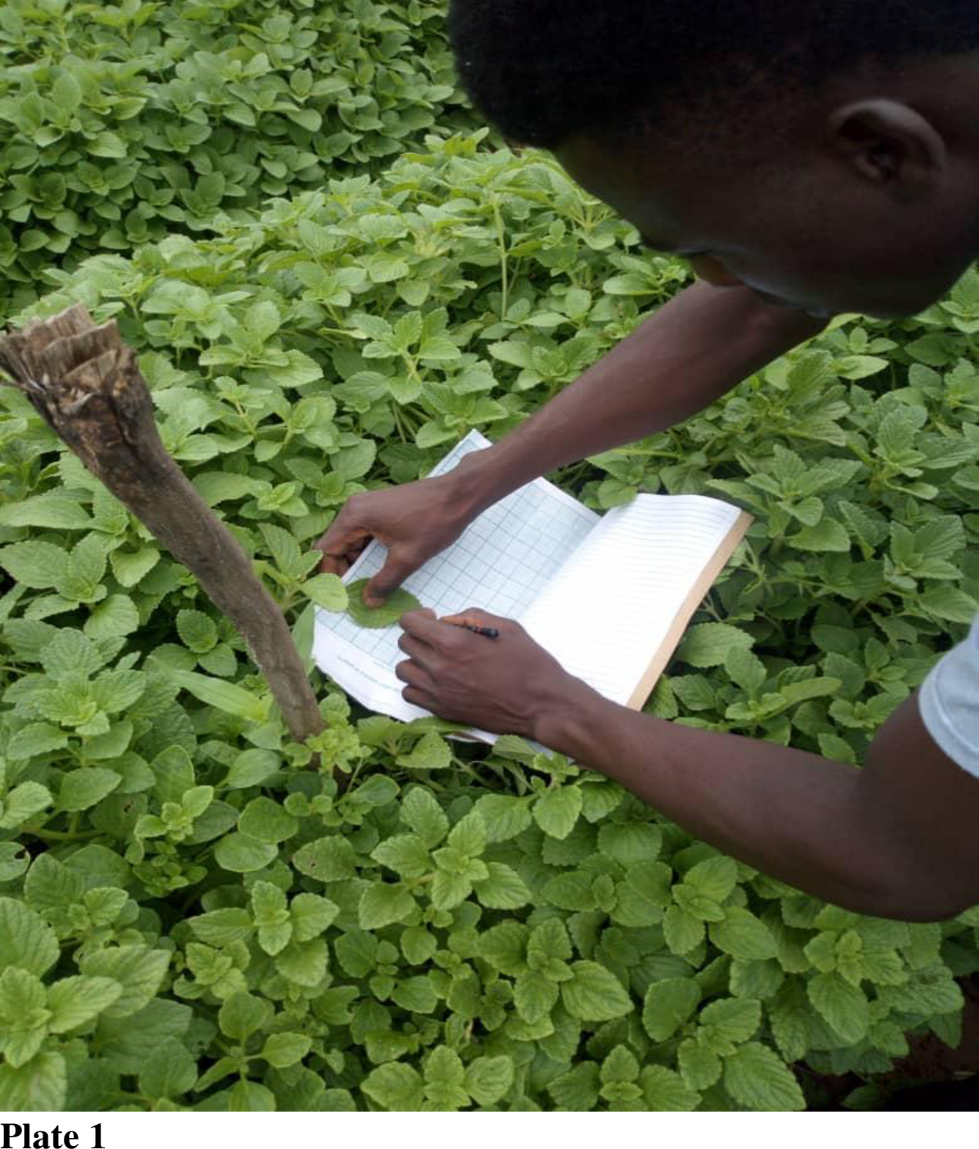


**Plate 1**

Supplement: S1 Fig — (DOCX) [file pone.0276566.s001.docx]

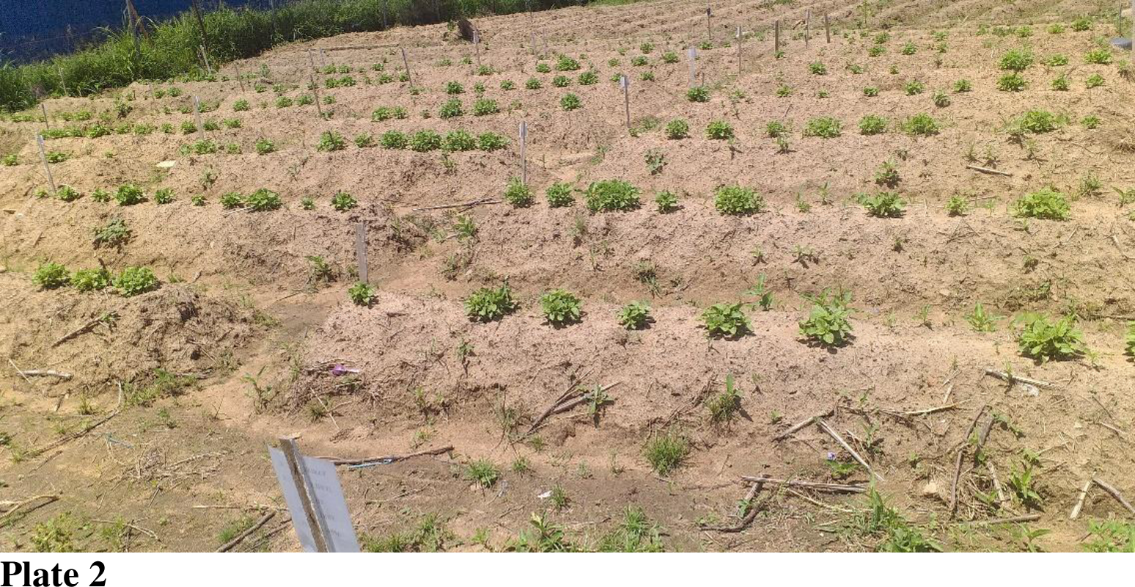


**Plate 2**

Supplement: S2 Fig — (DOCX) [file pone.0276566.s002.docx]

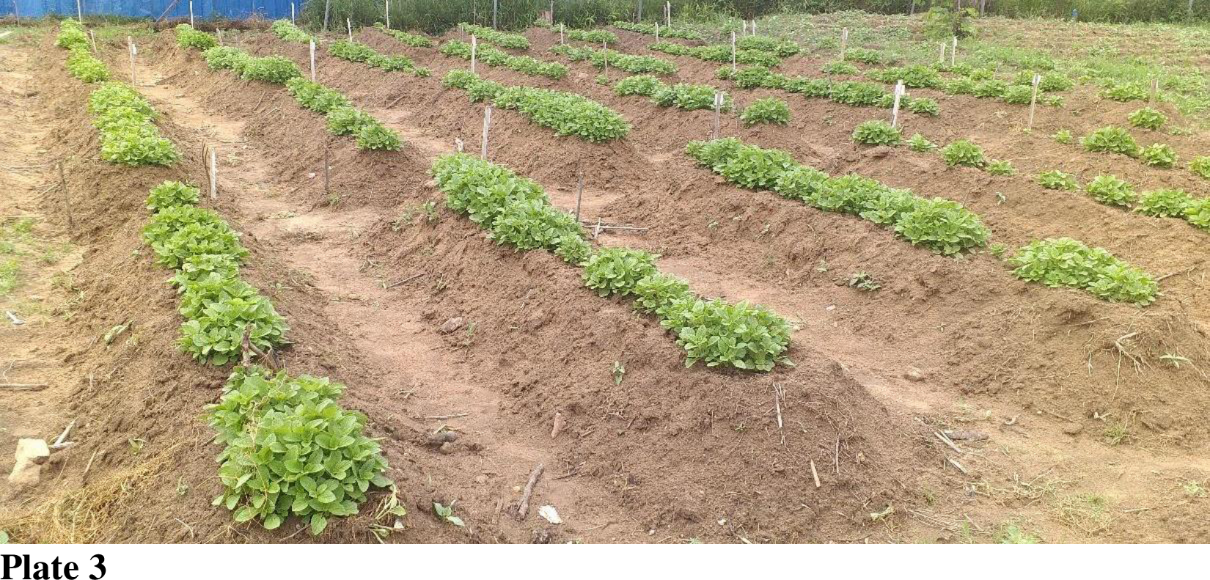


**Plate 3**

Supplement: S3 Fig — (DOCX) [file pone.0276566.s003.docx]

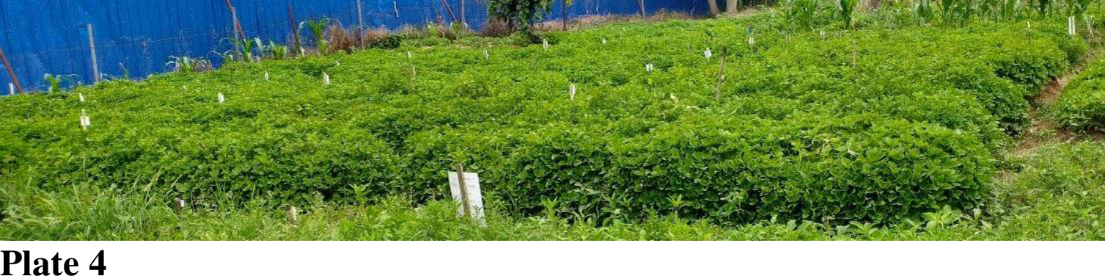


**Plate 4**

Supplement: S4 Fig — (DOCX) [file pone.0276566.s004.docx]

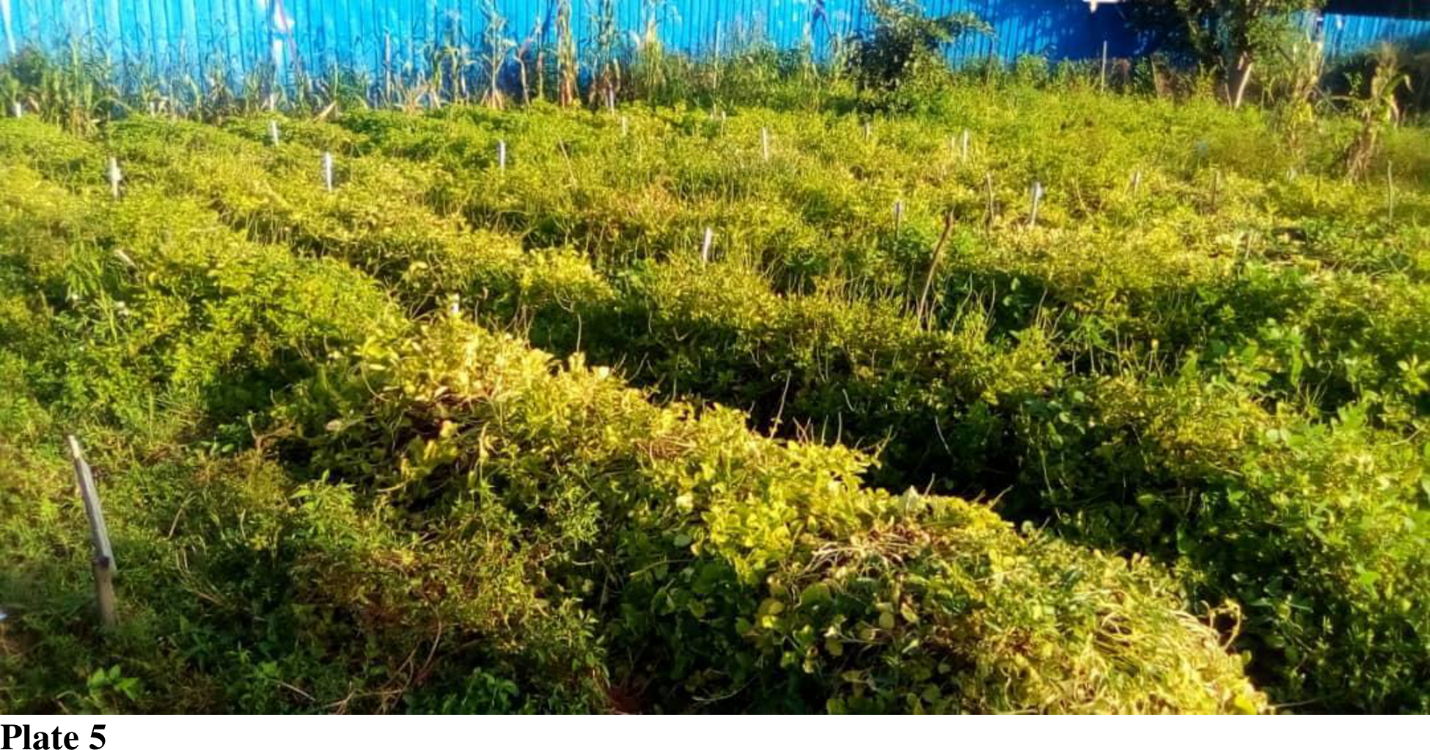


**Plate 5**

Supplement: S5 Fig — (DOCX) [file pone.0276566.s005.docx]

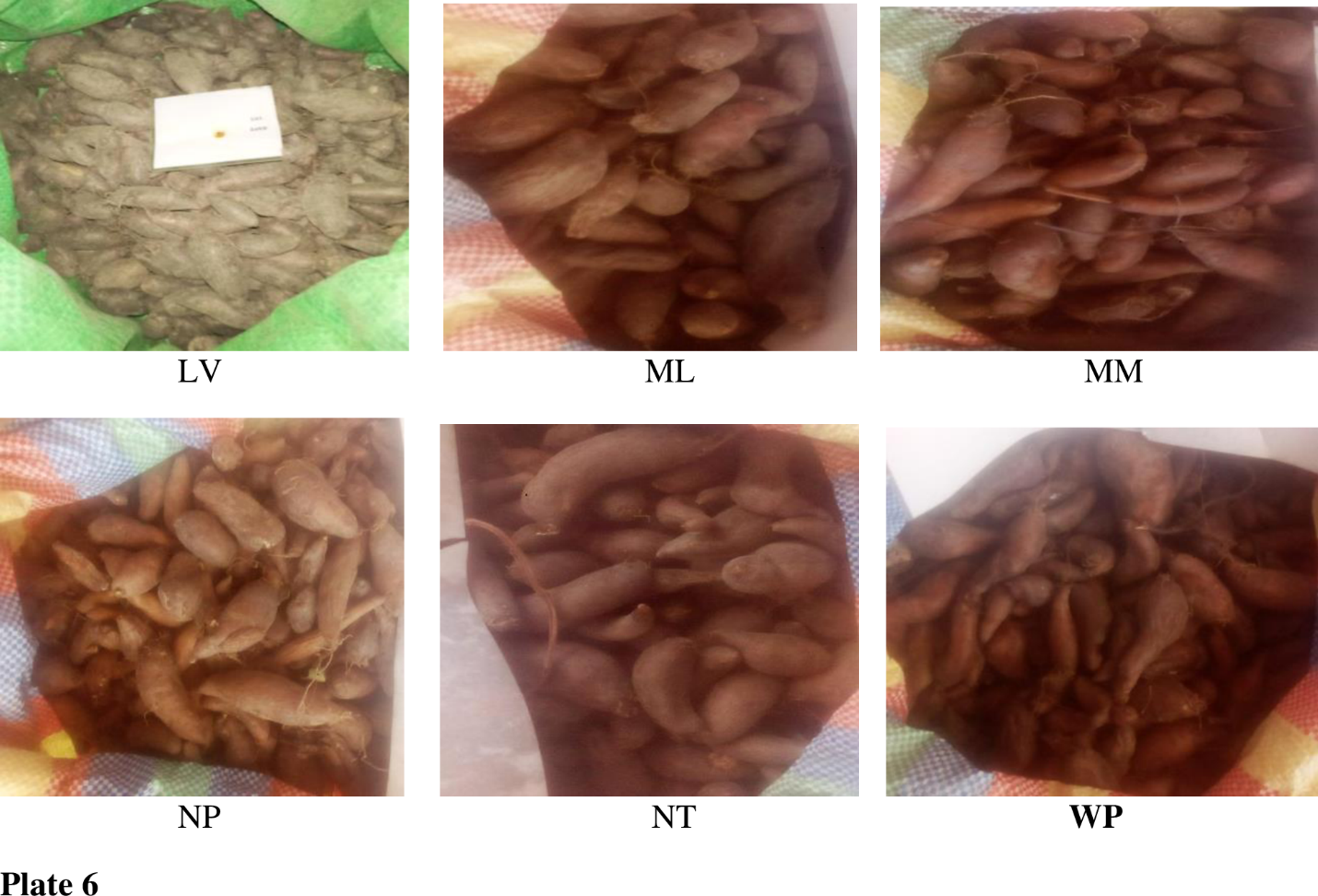


**Plate 6**

Supplement: S6 Fig — LV = Local variety, ML = Maa-Lana, MM = Manga-Moya, NP = Nutsuga Peisa, NT = Nachim–Tiir, WP = WAAP Peisa. (DOCX) [file pone.0276566.s006.docx]
