# Supplementary material for: Comparing the growth and yield performance of six different varieties of frafra potato (Solenostemon rotundifluis Poir) grown under rain-fed conditions in the Guinea Savanna ecological zone of Ghana
Source: PLoS One. 2022 Nov 16;17(11):e0276566. doi: 10.1371/journal.pone.0276566 (PMC9668201; doi:10.1371/journal.pone.0276566)
Supplement: Attachment — Submitted filename: PONE-D-22-14687_ last reviewed.pdf [file pone.0276566.s007.pdf]

PLOS ONE

Comparing the Growth and Yield Performance of Six Varieties of Frafra Potato (Solenostemon rotundifluis Poir) Grown under rain-fed conditions in the Guinea Savanna Ecological Zone of Ghana

--Manuscript Draft--

**Manuscript Number:**	PONE-D-22-14687
**Article Type:**	Research Article
**Full Title:**	Comparing the Growth and Yield Performance of Six Varieties of Frafra Potato (Solenostemon rotundifluis Poir) Grown under rain-fed conditions in the Guinea Savanna Ecological Zone of Ghana
**Short Title:**	Growth and Yield Performance of six varieties of Solenostemon rotundifluis Poir in Ghana.
**Corresponding Author:**	Abonuusum Ayimbire, Ph. D.<br>Bolgatanga Technical University<br>Bolgatanga, Ghana GHANA
**Keywords:**	Frafra potato; varieties; sprouted tubers; planting; ridges; growth parameters; leaf area; leaf length; petiole height; vine branches; plant height; canopy spread; yield; vegetative biomass
**Abstract:**	Aim: The study compared growth and yield performance of six varieties of Frafra potato under rain-fed conditions to help guide farmers in selecting high yielding varieties that grow well in deprived soils to improve household nutrition, income and food security.<br>Methodology: Field experiments were conducted for two consecutive rainy seasons using sprouted tubers in Randomized Complete Ridge Design (RCRD) with four replications. Each ridge measured 0.5 m high, 0.7 m wide and 3 m long, covering 2.1 m^2 of land. Data collected fortnightly was analyzed using STATA 16.0.<br>Results: The mean leaf area and plant height were 19.6 ± 6.8 cm^2 and 27.2 ± 10.0 cm respectively. The Local Variety had the highest leaf area (21.2 cm^2) while Manga-Moya recorded least leaf area (17.4 cm^2). Plants of WAAP Peisa were tallest (31.7 cm) as those of Local Variety were shortest (25.0 cm). Mean number of vine branches and canopy spread were 70 ±39 and 27.9 ± 9.4 cm respectively. The highest number of vine branches (88±56.7) was recorded in WAAP Peisa with the lowest of 57±33.1 in the Nachim –Tiir variety. The widest canopy of 30.6 ± 11.1 cm was recorded in Manga-Moya, while the narrowest canopy (25.1± 7.9 cm) was found in the Local Variety. Mean tuber yield was 20.7 ± 5.4 tons per hectare (t/ha). Local Variety produced highest tuber yield (27.8 ±4.7 t/ha) as Manga-Moya recorded lowest tuber yield (15.1 ±6.4 t/ha). Leaf area correlated positively with plant height (r = 0.78), canopy spread (r = 0.81) and number of vine branches (r = 0.70), but negatively with tuber yield (r = -0.01).<br>Conclusion: Local Variety produced lowest vegetative biomass but highest tuber yield. Manga-Moya gathered highest biomass but produced lowest tuber yield. Research and inputs can enhance potential of Frafra potato to reduce global hunger.
**Order of Authors:**	Abonuusum Ayimbire, Ph. D.
	Joseph Kunansua Laary, Ph. D.
	James Anaba Akolgo, Mphil
	Augustus Dery Ninfaa, Ph. D.
	Joseph Asampana Akolgo, Mphil
	Abdul Ganiu Anyagri Ndeogo, HND
**Additional Information:**	
**Question**	**Response**
**Financial Disclosure**	The author(s) received no specific funding for this work.

Powered by Editorial Manager® and ProduXion Manager® from Aries Systems Corporation

Enter a financial disclosure statement that describes the sources of funding for the work included in this submission. Review the submission guidelines for detailed requirements. View published research articles from *PLOS ONE* for specific examples.<br><br>This statement is required for submission and **will appear in the published article** if the submission is accepted. Please make sure it is accurate.<br><br>**Unfunded studies**<br>Enter: *The author(s) received no specific funding for this work.*<br><br>**Funded studies**<br>Enter a statement with the following details:<br>• Initials of the authors who received each award<br>• Grant numbers awarded to each author<br>• The full name of each funder<br>• URL of each funder website<br>• Did the sponsors or funders play any role in the study design, data collection and analysis, decision to publish, or preparation of the manuscript?<br>• **NO** - Include this sentence at the end of your statement: *The funders had no role in study design, data collection and analysis, decision to publish, or preparation of the manuscript.*<br>• **YES** - Specify the role(s) played.<br><br>* typeset	
**Competing Interests**<br><br>Use the instructions below to enter a competing interest statement for this submission. On behalf of all authors, disclose any competing interests that could be perceived to bias this work—acknowledging all financial support and any other relevant financial or non-financial competing interests.<br><br>This statement is required for submission and **will appear in the published article** if	The authors have declared that no competing interests exist.

Powered by Editorial Manager® and ProduXion Manager® from Aries Systems Corporation

the submission is accepted. Please make sure it is accurate and that any funding sources listed in your Funding Information later in the submission form are also declared in your Financial Disclosure statement.<br><br>View published research articles from *PLOS ONE* for specific examples.<br><br>**NO authors have competing interests**<br><br>Enter: *The authors have declared that no competing interests exist.*<br><br>**Authors with competing interests**<br><br>Enter competing interest details beginning with this statement:<br><br>*I have read the journal's policy and the authors of this manuscript have the following competing interests: [insert competing interests here]*<br><br>* typeset	
**Ethics Statement**<br><br>Enter an ethics statement for this submission. This statement is required if the study involved:<br><br>• Human participants<br>• Human specimens or tissue<br>• Vertebrate animals or cephalopods<br>• Vertebrate embryos or tissues<br>• Field research<br><br>Write "N/A" if the submission does not require an ethics statement.<br><br>General guidance is provided below. Consult the submission guidelines for detailed instructions. **Make sure that all information entered here is included in the Methods section of the manuscript.**	N/A

**Format for specific study types**<br><br>**Human Subject Research (involving human participants and/or tissue)**<br>• Give the name of the institutional review board or ethics committee that approved the study<br>• Include the approval number and/or a statement indicating approval of this research<br>• Indicate the form of consent obtained (written/oral) or the reason that consent was not obtained (e.g. the data were analyzed anonymously)<br><br>**Animal Research (involving vertebrate animals, embryos or tissues)**<br>• Provide the name of the Institutional Animal Care and Use Committee (IACUC) or other relevant ethics board that reviewed the study protocol, and indicate whether they approved this research or granted a formal waiver of ethical approval<br>• Include an approval number if one was obtained<br>• If the study involved *non-human primates*, add *additional details* about animal welfare and steps taken to ameliorate suffering<br>• If anesthesia, euthanasia, or any kind of animal sacrifice is part of the study, include briefly which substances and/or methods were applied<br><br>**Field Research**<br><br>Include the following details if this study involves the collection of plant, animal, or other materials from a natural setting:<br>• Field permit number<br>• Name of the institution or relevant body that granted permission	
**Data Availability**<br><br>Authors are required to make all data underlying the findings described fully available, without restriction, and from the time of publication. PLOS allows rare exceptions to address legal and ethical concerns. See the PLOS Data Policy and FAQ for detailed information.	No - some restrictions will apply

Powered by Editorial Manager® and ProduXion Manager® from Aries Systems Corporation

A Data Availability Statement describing where the data can be found is required at submission. Your answers to this question constitute the Data Availability Statement and **will be published in the article**, if accepted.<br><br>**Important:** Stating 'data available on request from the author' is not sufficient. If your data are only available upon request, select 'No' for the first question and explain your exceptional situation in the text box.<br><br>Do the authors confirm that all data underlying the findings described in their manuscript are fully available without restriction?	
**Describe where the data may be found in full sentences. If you are copying our sample text, replace any instances of XXX with the appropriate details.**<br><br>• If the data are **held or will be held in a public repository**, include URLs, accession numbers or DOIs. If this information will only be available after acceptance, indicate this by ticking the box below. For example: *All XXX files are available from the XXX database (accession number(s) XXX, XXX.*).<br>• If the data are all contained **within the manuscript and/or Supporting Information files**, enter the following: *All relevant data are within the manuscript and its Supporting Information files.*<br>• If neither of these applies but you are able to provide **details of access elsewhere**, with or without limitations, please do so. For example:<br><br>*Data cannot be shared publicly because of [XXX]. Data are available from the XXX Institutional Data Access / Ethics Committee (contact via XXX) for researchers who meet the criteria for access to confidential data.*<br><br>*The data underlying the results presented in the study are available from (include the name of the third party*	Please, all relevant data are within the manuscript and its Supporting Information files. However, I as corresponding author is able to provide any original data relating to the manuscript, like results of soil analysis from KNUST on request

and contact information or URL).<br>• This text is appropriate if the data are owned by a third party and authors do not have permission to share the data.<br><br>* typeset	
Additional data availability information:	

Manuscript Click here to access/download;Manuscript;Manuscript.docx

Comparing the Growth and Yield Performance of Six Varieties of Frafra Potato (*Solenostemon rotundifluis Poir*) Grown under rain-fed conditions in the Guinea Savanna Ecological Zone of Ghana.

Growth and Yield Performance of six varieties of *Solenostemon rotundifluis Poir* in Ghana.

Abonuusum Ayimbire[1] (aayimbire@bolgatu.edu.gh), Joseph Kunansua Laary[1] (jlaary@yahoo.co.uk), James Anaba Akolgo[1] (akonaj@gmail.com), Augustus Dery Ninfaa[1] (aninfaa@yahoo.com), Joseph Asampana Akolgo[2] (akolgo0211@yahoo.com), Abdul Ganiu Anyagri Ndeogo[1] (ndeogoanyagri@gmail.com).

Corresponding author: Abonuusum Ayimbire (aayimbire@bolgatu.edu.gh; 00233244972819).

[1]Department of Ecological Agriculture, School of Agriculture, Bolgatanga Technical University (BTU), P. O. Box 767, Bolgatanga, Ghana.

[2] Northern Development Authority, P. O. Box TL883 Industrial Area, Lamashegu, Tamale, Ghana.

Authors' contribution:

This work was carried out in collaboration among all authors. Authors AA and LJK designed the study, performed the soil analysis, wrote the protocol and AA wrote the first draft of the manuscript which was reviewed by authors LJK and AJA. Authors AJA and NDA supervised author NAAG to grow the crops and managed the analyses of the study. Author JAA carried out statistical analysis and interpretation of data. All authors read and approved the final manuscript.

How to cite this paper:

Ayimbire A, Laary JK, Akolgo JA, Ninfaa DA, Asampana JA, Ndeogo AAG. Comparing the Growth and Yield Performance of Six Varieties of Frafra Potato (*Solenostemon rotundifluis Poir*) Grown under rain-fed conditions in the Guinea Savanna Ecological Zone of Ghana. PLOS ONE….

Name of research group: BTU Research Group 2015

Abstract

Aim: The study compared growth and yield performance of six varieties of Frafra potato under rain-fed conditions to help guide farmers in selecting high yielding varieties that grow well in deprived soils to improve household nutrition, income and food security.

Methodology: Field experiments were conducted for two consecutive rainy seasons using sprouted tubers in Randomized Complete Ridge Design (RCRD) with four replications. Each ridge measured 0.5 m high, 0.7 m wide and 3 m long, covering 2.1 m^2 of land. Data collected fortnightly was analyzed using STATA 16.0.

Results: The mean leaf area and plant height were 19.6 ± 6.8 cm^2 and 27.2 ± 10.0 cm respectively. The Local Variety had the highest leaf area (21.2 cm^2) while Manga-Moya recorded least leaf area (17.4 cm^2). Plants of WAAP Peisa were tallest (31.7 cm) as those of Local Variety were shortest (25.0 cm). Mean number of vine branches and canopy spread were 70 ±39 and 27.9 ± 9.4 cm respectively. The highest number of vine branches (88±56.7) was recorded in WAAP Peisa with the lowest of 57±33.1 in the Nachim –Tiir variety. The widest canopy of 30.6 ± 11.1 cm was recorded in Manga-Moya, while the narrowest canopy (25.1± 7.9 cm) was found in the Local Variety. Mean tuber yield was 20.7 ± 5.4 tons per hectare (t/ha). Local Variety produced highest tuber yield (27.8 ±4.7 t/ha) as Manga-Moya recorded lowest tuber yield (15.1 ±6.4 t/ha). Leaf area correlated positively with plant height (r = 0.78), canopy spread (r = 0.81) and number of vine branches (r = 0.70), but negatively with tuber yield (r = -0.01).

Conclusion: Local Variety produced lowest vegetative biomass but highest tuber yield. Manga-Moya gathered highest biomass but produced lowest tuber yield. Research and inputs can enhance potential of Frafra potato to reduce global hunger.

1.0 Introduction

Frafra potato (*Solenostemon rotundifolius*) is a root-tuber crop of the *Lamiaceae* family, which is widely distributed and cultivated across the tropical African and Asian countries (Sugri *et al*., 2013; Enyiukwu *et al*., 2014; PROTA 2021). The crop has a multiplicity of names among different ethnic groups across these African and Asian countries, making it more difficult to collect reliable data and harmonize existing literature on the crop (Enyiukwu *et al*., 2014). In Ghana, the crop is mainly grown for food and income, and its production is restricted to the Guinea and Sudan Savanna vegetation zones, covering communities and regions of Northern Ghana (Sugri *et al*., 2013; Tortoea *et al*. 2020) bordering Togo and Burkina Faso. It thrives well in the dry savanna areas partly because, it can withstand adverse weather conditions and other consequences of changes in climatic conditions, including drought and high temperatures. It is also not a fastidious crop but can be grown with minimum inputs and devotion, so that the farmer can have spare time and resources to spend on other crops. The crop, whose tubers mature within 4-6 months, depending on the variety and other factors, can be harvested at leisure after maturity. However, the fleshy plant with delicate vine branches and leaves completes its life cycle within a year (Alleman and Hammes, 2006; Sugri *et al*., 2013).

The crop is reported to have the potential to grow to a height of about 15 cm, which can increase to 60 cm under favourable conditions. It also produces very small flowers whose colours range from pink, white, pale violet to blue, in a raceme-like inflorescence, which are hermaphrodites (Enyiukwu *et al*., 2014). Though the crop is grown purposely for its underground tuberous roots, the vegetative parts can be processed as feed to ruminants, especially in the dry season. It produces ovoid-shaped tubers that are highly rich in nutrients, minerals, vitamins and phytochemicals (Hua *et al*., 2018; Kwarteng *et al*., 2018; Nyawudzo *et al*., 2019). The tubers can be left on the farm in dry conditions for up to a year without tuber spoilage especially in the dry season. The crop has

some medicinal properties, hence the name '*Coleus dysentericus*' given to it in some areas suggests that it relieves patients off dysentery (Tindall, 1983; Sugri *et al*., 2013; Enyiukwu *et al*., 2014).

These and other qualities put Frafra potato among the category of crops that can be produced by an average subsistence farmer and can contribute to household food security in communities where it is cultivated (Hua, *et al*., 2018; Nyawudzo *et al*., 2019). The crop therefore, has great capability of becoming a commercial crop that can leverage the economic situations of subsistent farmers, thereby impacting lives and enhancing food security (Sugri *et al*., 2013).

Even though Frafra potato is touted as being economically viable and nutritionally fortified for rural households, information on the crop's potential is probably not well packaged and presented to local farmers and the general public. In consequence, the crop is neglected in terms of research focus. Hence, its cultivation is restricted within the indigenous rural communities, almost at the verge of extinction (Hua, *et al*., 2018; Kwarteng *et al*., 2018; Nyawudzo *et al*., 2019). Besides, improved varieties are either uncommon or unknown to local farmers (Sugri *et al*., 2013; Hua *et al*., 2018; Tortoea *et al*., 2020; PROTA 2021) ! Again, people generally discriminate against Frafra potato production and consumption using factors of convenience (tuber size for example) rather than those of necessity (nutrient fortification, for instance), to neglect the crop which is contributing to its production decline (Sugri *et al*., 2013).

Therefore, research focused on improving tuber sizes, tuber yields and vegetative components of the crop, using available varieties is appropriate. Moreover, selecting promising varieties with the desirable characteristics in vegetative growth and tuber development for farmers, can draw the attention of local farmers and the general public, to enhance its cultivation and utilization.

Conducting studies on the existing varieties of the crop, under similar growth conditions, will help in comparing and identifying high yielding varieties and recommending same to farmers for cultivation. This will help improve the fortunes of farmers producing the crop, and will soar up production to enhance household food security, while safeguarding its extinction (Sugri *et al.*, 2013; Nyawudzo *et al.*, 2019). This study therefore aimed at evaluating the vegetative growth and root tuber yield performance of six Frafra potato varieties under rain-fed conditions, in the Upper East Region of Ghana. The findings of two varieties; one producing more root tuber yield with less vegetative growth and the other performing the vice versa is indicative of the achievement of this objective.

2.0 Methodology

2.1 Study Location

The study was conducted on the experimental field of the Department of Ecological Agriculture, Bolgatanga Technical University (BTU), Sumbrungu, located at 10.8296101°N, 0.9414592°W and within Bolgatanga Municipality (10.7875°N, 0.8580°W) in the Upper East Region of Ghana (Fig1.1).

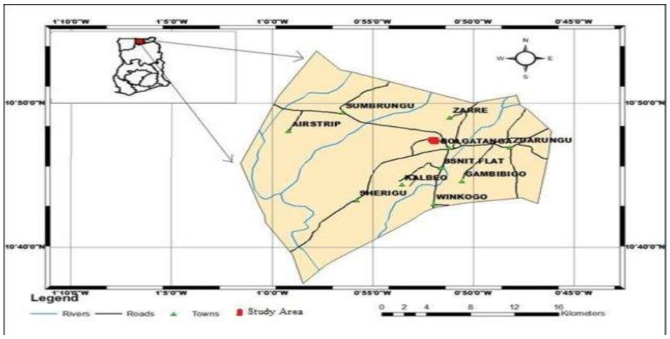

Fig 1.1: A Map of Bolgatanga Municipal (showing BTU, Sumbrungu) of the Upper East Region, and its location on Ghana map.

Bolgatanga Municipal is within Bolgatanga, the capital of the Upper East Region of Ghana. The region is located in the Sudan Savanna ecological zone of Ghana, with erratic rainfall distribution and pattern, averaging 800 mm from May to June and about 1,100 mm from September to October. The night average temperatures are about 14°C and about 35°C during the afternoon part of the day, with low relative humidity. The vegetation generally consists of short, widely dispersed deciduous trees and grasses which have adapted to the low moisture conditions during the long dry seasons (GSS, 2014).

2.2 Soil Sampling and Testing

The study was conducted in the rainy season with conditions similar to those of the local farmers, for a better assessment of the growth and tuber production of the crop and for subsequent recommendations to be made to farmers. Soils were sampled on two experimental sites adjacent to each other, at 20 cm depth and analyzed for some properties before planting and after harvesting to assess the soil capacity to support Frafra potato crop production, following Taylor and Francis (2008) and Fery *et al*. (2018).

Using a hand probe, three (3) soil samples (0-20cm depth) were taken at about equal intervals, from the top of each of the four (4) replicate ridges in each experimental site. Collected soil samples were put into clean, black plastic bags. Thus, there were twelve (12) soil samples per replication (3 samples per ridge by 4 replicate ridges), given seventy-two (72) soil samples. Each set of twelve (12) soil samples from each replication was poured into a clean bucket, well-mixed and a composite sample of about 30 g parceled, appropriately labelled and analyzed at the Crop and Soil Sciences laboratory of Kwame Nkrumah University of Science and Technology, Kumasi, Ghana. The soil samples were also taken after harvest, using the same procedure and analyzed at the same laboratory.

2.3 Land Preparation, Field Layout and Planting

The experiment was conducted in the rainy seasons from May-November in 2020 and 2021, using six different Frafra potato varieties, in a randomized complete Block Design (RCBD) with four replications. These six varieties were Manga-Moya (MM), Maa-Lana (ML), Nutsuga Peisa (NP), Nachim –Tiir (NT), WAAP Peisa (WP) and Local Variety (LV).

In both rainy seasons adjacent fields were used for the experiment and were cleared of debris and ridges prepared using hoe to soften the land before planting. Each ridge measured 0.5 m high, 0.7 m wide and 3 m long. The holes were created with a hoe and sprouted tubers carefully planted at the onset of rains in May, 2020 and 2021. Sprouted seedlings were gently covered with topsoil to avoid breaking the sprouted delicate shoot or burying them in the soil. Ridges are suitable for the tuberous root formation (Dumbuya *et al*., 2016), and tuber yield is likely to be maximized when the sprouted tubers are planted between May and the middle of June (Sugri *et al*., 2013).

2.4 Cultural Practices and Pest Management

The experiment was conducted in the rainy seasons of 2020 and 2021, but plants were sparingly watered at the beginning prior to onset of rains. The field was weeded thrice after planting using hoe. During weeding, soil was raised with hoe to support the base of the plants to prepare them for shooting of pods for tuber formation. Weeds were also occasionally pulled out by hand.

The foliage of the plants were regularly inspected for incidence of pests and diseases using magnifying glasses. The insect pests, such as aphids, white flies, grasshoppers and caterpillar-like larval forms were occasionally found and identified on the vegetative parts of the Frafra potato crop. However, no pesticide was sprayed on the crops because, the pests were not at threshold levels to cause appreciable damage or change on the growth and yield performance of the crop.

Farmers generally do not experience or consider pests as a threat to Frafra potato crop, whose leaves are usually not the desired choice of most herbivorous animals (Sugri *at al*. (2013).

2.5 Measurement of Growth Parameters

Data collection, which was done fortnightly, started a month (about 4 weeks) after planting in both rainy seasons. Four (4) plants were randomly selected per variety (a plant per replication) for data measurement in each experiment. Thus, data on growth parameters was collected from twenty four (24) plants in each experiment. Each selected plant was marked by loosely tying a coloured plastic band to one of its branches for easy identification. The following growth parameters were measured: Plant height (cm), Leaf area (cm^2), number of vines and canopy spread (cm). The mass of tubers (Kg) produced by each variety of Frafra potato was also measured.

2.6 Estimation of Leaf Area [cm^2]

To estimate the leaf area, a leaf was randomly selected from the randomly selected plants per variety. The selected leaf was labelled by tying it loosely with a coloured plastic band. In all, four (4) leaves were labelled per treatment to give a total of twenty-four (24) leaves for the six (6) treatments.

Without plucking the selected leaf from the plant, the leaf area was measured by tracing the outline of the selected leaf blade on a graph sheet using a pencil (Plate 4). The leaf area was estimated by counting the number of one centimetre squares (1 cm^2) that each leaf outline covered. Suppose the number of 1 cm^2 fully covered by the leaf outline was counted to be x_1. Where at least three-quarters of the squares were covered by the leaf outline, they were counted as being fully covered, which is x_2. Then, the number of 1 cm^2 that were half-covered by the outline were counted to be y,

which was divided by two to obtain full 1 cm^2 that is y/2. Where at most a quarter of squares were covered by the leaf outline, these were not counted. Therefore the total estimated area of the leaf (cm^2) was obtained by adding the number of 1 cm^2 that were fully covered by the leaf outline ($X_1 + X_2 + Y/2$).

Plate 4: Tracing the outline of a leaf on a graph in order to estimate its area.

2.7 Vine Branches and Canopy Spread [cm]

The number of vine branches produced by the selected plants per variety were manually counted at regular interval of days (fortnightly) and recorded. The vine branches make up the canopy, and the canopy spread was determined using a centimeter rule to measure the radius of the crown of the selected plants. This was obtained by taking the diameter of the crown in two different directions and average values recorded. This average diameter was then divided by two to obtain the radius as the canopy spread (cm).

2.8 Days to Flowering and Maturity

Days to flowering were determined by observing and recording the dates on which flowering began. The number of days from planting to flowering was determined by comparing the planting date with the date of flower initiation. The days to maturity was also obtained by comparing the date of planting with the date of tuber maturity. The tubers were harvested after maturity.

2.9 Tuber Mass [kg]

The Frafra potato tubers were harvested at maturity on per plant per variety bases. Tubers of each variety per replicate ridge were harvested separately and tubers weighed (in Kg) using a beam balance. Thus, the mass of the tubers of each variety per experiment was obtained by summing the mass of tubers of that variety in the replicates and averaging.

2.10 Rainfall Data

Rainfall data within the Bolgatanga Municipality, covering the period the Frafra potato was cultivated, from April to November, 2020 and 2021, was obtained from the Ghana Meteorological Agency, Bolgatanga, Upper East Region. Though distribution differed in both season, the rainfall amounts were without statistical differences and the average values were used.

2.11 Data Analysis

The data sets were analyzed using statistical programme, Stata 16.0, StataCorp LLC, College Station, Texas 77845, USA. Comparison of Frafra potato varieties with respect to any measured parameter was at the 5% level of confidence. The analyzed data sets in both experiments were not significantly different and were combined and analyzed to obtain the results.

3.0 Results and Discussion

3.1 Soil Properties of the Study Site

The analyzed soil results (Table 3.1) show that the soil texture of the study site is mainly sandy loamy, with the proportion of sand being 84.7% before planting which decreased to 84.0% after harvesting. The composition of silt decreased from 6.4% before planting to 5.7% after harvesting. On the contrary, the percentage of clay increased from 8.9% to 10.3% after harvesting.

Table 3.1: Soil structure, chemical reaction and nutrient content of the study site

	Soil sample mean values	
**Soil parameter**	Before planting	After planting
**Soil structure**		
Sand, %	84.70	84.0
Silt, %	8.94	10.3
Clay, %	6.36	5.74

Texture class	Loamy sand	Loamy sand
**Organic components**		
Organic Carbon, %	0.34	0.33
Organic matter, %	0.59	0.57
% Total N	0.13	0.12
Avail. P, mg/kg	21.77	17.51
pH	6.62	5.77
**Exchangeable acidity**		
Al, cmol/kg	0.59	0.59
H, cmol/kg	0.32	0.23
**Exchangeable cations**		
K, cmol/kg	0.65	0.31
Ca, cmol/kg	3.2	2.98
Mg, cmol/kg	1.65	1.03
Na, cmol/kg	0.05	0.03
**Micronutrients**		
Fe, mg/kg	10.86	17.13
Zn, mg/kg	1.27	1.45
Mn, mg/kg	15.40	21.16
Cu, mg/kg	0.69	1.42

The fraction of silt (1.60%) remained about the same before planting and after harvesting. The higher proportion of sand makes the soil particles loose, less adhesive and less compacting, making the soil suitable for root penetration and tuber formation. The soil with small proportions of silt and clay relative to sand are usually well-draining, a good requirement of Frafra potatoes for maximum tuber yield (Sugri *et al*., 2013), despite its low moisture retention capacity.

The soil was also found to be slightly acidic with an average pH value of 6.6 before planting, decreasing to a pH value of 5.8 after harvesting. Before planting, the soil exchangeable acidity (Al^{3+} and H^{+} concentrations) was 0.59 cmol/kg of Al^{3+} and 0.32 cmol/kg H^{+}. Whereas, after harvesting, the H^{+} concentration decreased to 0.23 cmol/kg, that of the Al^{3+} remained unchanged, at 0.59 cmol/kg (Table 3.1). This implies that Frafra potato crop could have facilitated the removal of H^{+} ions from the soil and may perform better in acidic soils. In the case of sweet potato crop, it facilitated the release of H^{+} ions to the soil and increase soil pH (Abonuusum *et al*., 2021).

Soil pH and exchangeable acidity determine the solubility and availability of certain nutrients for plants to use, because whereas some nutrients are soluble and available in acidic soils, others dissolve and are available in alkaline or basic soils (Motsara et al., 2008).

The contents of organic matter and organic carbon were low in the study soil before and after harvest of corps of the varieties. Among the trace elements tested, manganese level was 15.4 mg/kg before planting and increased to 21.2 mg/kg after harvesting.

The total nitrogen (N) content before planting was 0.13% and after harvesting was 0.12%N, suggesting Frafra potato crop may sparingly utilize soil nitrogen for its growth and tuber development, and can do well in nitrogen deficient soils. On the contrary, the available phosphorus content before planting was 21.8%, but decreased to 17.5% after harvesting, suggesting that Frafra potato make more use of soil phosphorus for its growth and development.

The soil of the experimental site had limited concentration of nutrients, and may require fertilizer or nutrient augmentation to optimize crop yield on the site. Though Frafra potato may sparingly mine soil nutrients for its growth and development, the crop require adequate amounts of soil nutrients, especially phosphorus, for its production. There is therefore the need for local farmers to improve soil nutrients status of their fields for frafra potato production to maximize tuber yields.

3.2 Growth and development characteristics of Frafra potato

The mean values of the measured growth and development parameters are presented in Table 3.2. Photos of the growth and development stages of the frafra potato plants are presented in plates 1 – 5. There were variations in vegetative growth (plant height, leaf area, canopy spread, vine number) and reproductive growth (days to flowering, days to maturity and tuber production) among the six Frafra potato varieties.site here like (Plate 1, 2,3....) not all plate title like you presented.

Plate 1: The Frafra potatoes five weeks after planting

Plate 2: The Frafra potatoes seven weeks after planting

Plate 3: The Frafra potatoes three months after

Plate 4: Tracing the outline of a leaf on a graph in order to estimate its area

Plate 5: The Frafra potatoes at maturity

Table 3.2: Mean values of the growth and yield parameters of Frafra potato varieties

**Variety**	Plant height (cm)	Leaf Area (cm^2)	Canopy spread (cm)	Number of vines	Days to Flowering	Days to Maturity	Tuber yield (ton/h)
	Mean ± SD	Mean ± SD	Mean ± SD	Mean ±SD	Mean±SD	Mean±SD	Mean ±SD
LV	25.0 ±7.1^{a}	21.2±7.0^{b}	25.0±7.9^{a}	58.8±32.5^{a}	90±5.0b	138±1.3a	27.8±4.7^{c}
ML	25.4±6.8^{a}	18.3±7.7^{a}	27.6±8.6^{a}	66.0±33.1^{b}	85±5.1a	138±1.2a	19.3±2.7^{b}
MM	29±10.1^{b}	17.4±5.8^{a}	30.6±11.1^{b}	82.8±46.4^{b}	92±5.9b	145±1.4a	15.1±6.4^{a}
NP	25.9±7.4^{a}	18.7±6^{a}	28.0±9.9^{a}	63.1±32.8^{a}	93±2.7b	145±1.3a	20.8±6.3^{b}

NT	26.4±8.8^{a}	20.8±7.3^{b}	26.6±9.4^{a}	56.8±33.1^{a}	84±4.7a	138±1.1a	25.0±3.7^{c}
WP	31.7±13^{b}	20.3±6.6^{b}	29.6±9.2^{b}	88.2±56.7^{c}	89±7.4b	145±1.3a	16.6±3.5^{a}
**Mean**	**27.5±8.9**	**19.6±6.8**	**27.9±9.4**	**69.5±38.7**	**89±5.6**	**142±1.4**	**20.7±5.4**
**%CV**	**32.6**	**34.6**	**33.4**	**56.4**	**5.6**	**0.9**	**27.4**

FPV = Frafra Potato Variety; LV = Local variety, ML = Maa-Lana, MM = Manga-Moya, NP = Nutsuga Peisa, NT = Nachim –Tiir, WP = WAAP Peisa; SD = Standard deviation.

Values with the same superscript letter are not significantly different at 5% level of probability

3.3 Plant height [cm]

The mean plant height across all six varieties was 27.5 ± 13.8 cm. However, plant height varied from tallest of 31.7 ± 13 cm in WP variety to the shortest of 25 ± 7cm in LV. The MM and WP varieties were the tallest and were significantly different ($P<0.05$) in height from the rest of the varieties (Table 3.2). This indicates that there are variations in plant height (CV= 33%) among the Frafra Potato varieties.

Table 3.3 presents the mean correlations of growth and development parameters to each other and to yield. Plant height across varieties was highly positively correlated to leaf area (r = 0.78). The highest correlation of plant height to leaf area was recorded in the LV (r = 0.93) while the lowest (r = 0.73 was recorded in the NP variety (Table 3.3 b). This indicates a strong association between leaf development and plant height in Frafra potato, and suggests some level of dependency in growth and development among vegetative parts of the crop.

Table 3.3: Mean correlation of growth parameters to each other and to yield

	Plant height (cm)	Leaf area (cm^2)	Canopy spread (cm)	Number of vines	Days to flowering	Days to maturity	Yield
Plant height (cm)	1.00						
Leaf area (cm^2)	0.78	1.00					

Canopy spread (cm)	0.95	0.81	1.00				
Number of vines	0.93	0.70	0.93	1.00			
Days to flowering	-0.07	0.01	-0.02	-0.02	1.00		
Days to maturity	0.12	-0.10	0.11	0.17	0.40	1.00	
Yield	0.05	-0.01	0.02	-0.02	-0.77	-0.21	1.00

Table 3.3b: Correlation of growth parameters to each other and to yield in the varieties

**LV variety**	Plant height (cm)	Leaf area (cm^2)	Canopy spread (cm)	Number of vines	Days to flowering	Yield
Plant height (cm)	1.00					
Leaf area (cm^2)	0.93	1.00				
Canopy spread (cm)	0.98	0.94	1.00			
Number of vines	0.91	0.87	0.93	1.00		
Days to flowering	-0.05	-0.06	-0.05	-0.11	1.00	
Yield	0.06	0.0598	0.06	0.10	-0.99	1.00

ML variety

Plant height (cm)	1.00					
Leaf area (cm^2)	0.82	1.00				
Canopy spread (cm)	0.95	0.82	1.00			
Number of vines	0.92	0.74	0.96	1.00		
Days to flowering	0.06	-0.03	0.04	-0.02	1.00	
Yield	-0.07	0.02	-0.02	0.04	-0.94	1.00

MM variety

Plant height (cm)	1.00					
Leaf area (cm^2)	0.81	1.00				
Canopy spread (cm)	0.96	0.87	1.00			
Number of vines	0.94	0.79	0.96	1.00		
Days to flowering	-0.21	0.06	-0.10	-0.04	1.00	
Yield	0.21	-0.03	0.14	0.07	-0.92	1.00

NP variety

Plant height (cm)	1.00					
Leaf area (cm^2)	0.73	1.00				
Canopy spread (cm)	0.96	0.78	1.00			
Number of vines	0.91	0.74	0.95	1.00		
Days to flowering	-0.04	0.30	0.015	0.09	1.00	
Yield	0.06	-0.28	-0.003	-0.09	-0.99	1.00

NT variety

Plant height (cm)	1.00					
Leaf area (cm^2)	0.90	1.00				
Canopy spread (cm)	0.98	0.89	1.00			
Number of vines	0.93	0.78	0.95	1.00		
Days to flowering	0.014	-0.005	-0.05	-0.057	1.00	
Yield	-0.0005	0.03	0.027	0.0036	-0.86	1.00

WP variety

Plant height (cm)	1.00					
Leaf area (cm^2)	0.78	1.00				
Canopy spread (cm)	0.95	0.90	1.00			
Number of vines	0.95	0.75	0.93	1.00		
Days to flowering	-0.21	0.19	-0.12	-0.14	1.00	
Yield	0.17	-0.16	0.11	0.10	-0.96	1.00

The plant height also positively but weakly correlated to tuber yield in four (4) of the varieties ((LV: r = 0.06; MM: r = 0.21; NP: r = 0.06; WP: r = 0.17) and negatively correlated to tuber yield in two varieties (ML: r = - 0.067; NT: r = - 0.001) (Table 3.3 b). Similar weak correlation trends with tuber yield was found in leaf area, canopy spread and vine development (Table 3.3 b). These correlation values show that there is virtually no association between plant height or vegetative development and tuber yield, and that vegetative growth is not a determining factor for tuber development and yield in Frafra potato.

Other investigators have also recorded both positive and negative correlations between the length of vines and root tuber yield, albeit they worked on varieties of sweet potato. Ochieng (2019), recorded a positive link between vine internode length and root yield of sweet potatoes. Abonuusum et al. (2021) a found positive relationship between the vine length and mass of root tuber in three varieties of sweet potato in the range of r = 0.02 and r = 0.19 as well as negative correlations in two others in the range of r = -0.05 and r = -0.03.

3.4 Leaf area [cm^2] development

The mean per leaf area across varieties was 19.6 ± 6.8 cm^2. The highest per leaf area of 21.2±7 cm^2 was recorded in LV, while the least per leaf area of 17.4 cm^2 was recorded in MM. However

per leaf area values between MM, ML and NP were not significantly different; and that between WP, NT and LV were also not significantly different at 5% level of confidence. The differences in per leaf area values of latter three varieties, were significantly higher ($P < 0.05$) than the former.

In addition, the average standard deviation value of 6.8 across implies that, leaf area is widely spread among Frafra potato varieties. In other words there are variations (CV= 35%) in leaf expansion in Frafra potato. Given that leaf area is directly related to water loss through evapotranspiration, the local variety with the largest leaf area, might lose more water and will possibly require more water for its growth and development.

However, table 3.3 shows that per leaf area of the varieties is negatively correlated to the tuber yield ($r = -0.01$), which is an indication that tuber development is independent of leaf area and leaf size is not a yield determinant in Frafra potato. It does appear that leaf area is directly proportional to the vegetative organs of the plants. Therefore, increase in plants leaf area beyond a certain critical value, depending on the level of growth of the plant, promotes the growth of plants vegetative parts, by influencing the conduction of photosynthesized food to these parts, leading to increased vegetative biomass production at the expense of tuber yield (Fleck et al., 2012; Isa et al., 2015; Su *et al*., 2016; Widaryanto and Saitama, 2017; Abonuusum *et al*., 2021).

This may imply that by default, food synthesized in the leaves is more likely to be translocated to the growth of vegetative structures at the expense of tuber formation and development. This probably explains why plants under normal growth conditions tend to gather vegetative biomass from germination or sprouting, and only start to channel assimilates to reproductive parts during stress or reproductive phase.

3.5 Plant canopy spread [cm]

A mean plant canopy spread of 27.9±9 cm was recorded across all six varieties. These varieties though, appeared to be similar in stature, varied in canopy spread (CV=33%). The MM variety had the widest plant canopy spread of 30.6±11 cm, whereas the narrowest plants canopy spread of 25.1± 8 cm was recorded in LV variety. The canopy spread in WP and MM varieties were significantly (P< 0.05) higher than the other varieties (Table 3.2).

The canopy spread strongly correlated positively with leaf area, with the LV variety recording the highest value (r = 0.94) and the NP variety the least value (r = 0.78) (Table 3.3 b) These strong positive relationships suggests some high level of interdependency between leaf area and canopy spread in Frafra potato.

3.6 Vine branches

The average number of vine branches produced in all the six Frafra potato varieties was 70 ± 39. However, the highest number of vine branches per plant (88 ± 57) was recorded in WP, and the lowest number (57±33) in NT variety. There were generally very high variation (CV= 56%) in vine numbers across the six Frafra potato varieties, suggesting that the varieties are inherently different in vine development. There was however, a highly positive correlation (r = 0.70) between leaf area and vine branches formed in all the six varieties. The correlation was highest with the LV (r = 0.86) and lowest with both ML and NP (r = 0.74) (Tables 3.3, 3.3b). There is a higher possibility that, when the number of vine branches are more, more leaves will be produced.

Generally, the correlation between vegetative parts like plant height, canopy spread, vine branches and leaf area is in synchrony with physiology, because growth of these vegetative parts is

dependent on synthesized food from the leaves. Thus, the larger the leaves, the higher the surface area for trapping both carbon dioxide and sunlight for photosynthesis; and the greater the assimilate availability for vegetative biomass production. Additionally, the growth performance of these vegetative organs of Frafra potato could be determined by available soil water and nutrients, thereby influencing tuber formation.

3.7 Flowering and maturity periods

The days to flowering of the six varieties ranged from 84 ±5 days in NT variety to 93±3 days in NP variety, with an overall average of 89± 6.0 days (about 3 months) after planting. (Table 3.2). Though days to flowering is an important components of yield, there was a strong negative correlation (r = - 0.8) between days to flowering and tuber yield in Frafra potato, suggesting that early or late days to flowering does not necessarily translate to tuber development. It also indicates that flowering is not a determinant of tuber formation in Frafra potato.

In Frafra potato, the observable signs of tuber maturity are the yellowing of the leaves, withering and falling of the flowers as well as cracking on the ridges due to expanded tubers (Sugri *et al.*, 2013). All the six varieties had their tubers ready for harvest within an average period of 142 ± 1) days (about 5 months) after planting. Three of the varieties (LV, ML and NT) matured within 138 ±1 days after planting, while other three varieties (MM, NP and WP) matured within 145±1 days after planting.

This maturity period is in line with Kana *et al*. (2012) and Enyiukwu *et al*. (2014) that Frafra potatoes mature within 5-6 months after planting. Number of days to maturity correlated negatively with the tuber yield (r = - 0.21), indicating that tuber yield does not depend on the crop's maturity period. Hence, early or late maturing variety can give either good or poor yield.

3.8 Tuber yield

The mean tuber yield across the six Frafra potato varieties was 20.7 ± 5.4 tons per hectare (t/ha) (Table 3.2). There were variations (CV=27.4%) in tuber yield across the six varieties, indicating inherent differences of the varieties in tuber production. The highest tuber yield of 27.8 ±4.7 t/ha was recorded in the local variety (LV) and the lowest of 15.1 ± 6.4 t/ha was recorded in Manga-Moya (MM) variety. Though, The LV plants, which gathered the least vegetative biomass, being the shortest plants with the narrowest canopy spread and among those with the lowest number of vine branches. it recorded the highest leaf area and tuber yield. The large leaves probably produced more photosynthates and channeled same into more tuber formation than vegetative structures.

On the other hand, the MM variety which formed the largest vegetatve organs, being the tallest plants with the highest canopy spread and number of vine branches produced the smallest leaves and tuber yield. Generally, varieties with high vegetative biomass recorded relatively lower tuber yields (plates 4 - 6), indicating assimilates were channeled into vegetative development at the expense of tuber formation. These observations are supported by the positive correlation between yield and leaf area of varieties of plants which have comparatively large leaves but are short, have small number of vine branches and narrow canopies (LV: r = 0.06; ML: r = 0.02; NT: r = 0.03). On the contrary, the correlation between yield and leaf area of plants of varieties with small leaves which are comparatively tall, have large number of vine branches and wide canopies is negative (MM: r = -0.03; NP: r = -0.28; WP: r = -0.16). This findings corroborate reports that leaf area is directly related to the growth of plant vegetative organs or root tubers (Fleck *et al.*, 2012; Isa *et al.*, 2015; Su *et al.*, 2016; Widaryanto and Saitama, 2017; Abonuusum *et al.*, 2021).

Plate 6: Tubers of the studied six varieties of Frafra potato.

The tuber yield of the six varieties in this study under the rainfall condition, without addition of manure or fertilizer is encouraging. This was higher than the projected 5-15 t/ha under favourable growth conditions (Tortoea *et al*., 2020), albeit much lower values than the postulated 45 MT/Ha (Nkansah, 2004; Enyiukwu *et al*., 2014). It is therefore obvious that very high yield of Frafra potatoes is achievable at the study site, under optimum growth conditions.

3.9 Rainfall and leaf expansion in Frafra potato

Leaves of the six Frafra potato varieties expanded steadily from four (4) weeks after planting (WAP) until 12 WAP (Fig 3.1). With the exception of the Local variety (LV), the leaves of other varieties probably reached their maximum sizes and stopped expanding 12 WAP. Leaf area of the LV, Manga-Moya (MM) and Maa Lana (ML) increased consecutively from 4-8 WAP (Fig3.1).

Fig 2.1: Rainfall and leaf area expansion in Frafra potato

Though there was a decrease in rainfall by the 8th WAP, and consequent decline in leaf growth; the rate of increase in leaf area within 4-8 WAP was higher than from 10-12 WAP, when rains were rather heavier (Fig 3.1). The reduction in the leaf expansion within that growth phase could be due to plants approaching maturity, and leaves attaining their maximum sizes or due to too much soil moisture (Enyiukwu *et al*., 2014).

The MM variety however did not recover from the mild drought and continued at a reduced rate of growth till maturity. The other five varieties recovered and continue to grow, though at varying rates, till maturity at 14 WAP (Fig 3.1) and may be considered as being drought-resistant to varying degrees (Safwan and Mohammed, 2016; Nyawudzo *et al*., 2019).

3.10 Rainfall and plant height in Frafra potato

The six varieties progressively grew taller with time, from 4-10 WAP (Fig 3.2). However, the WAAP Piesa (WP) variety grew vertically fastest as compared with LV and ML varieties (Fig. 3.2).

Fig 2. 2: Rainfall and plant height in Frafra potato

Though the rainfall decreased at the 8th WAP, that did not appear to have much influence on the vertical growth of the varieties studied. This shows that moderate rainfall could suffice for the growth and development of Frafra potato crops. However, there was increased vertical growth especially in WP and Manga-Moya (MM) variety, during heavy rains at the 10th WAP.

Plant height of the Nutsuga Piesa (NP), Nachim –Tiir (NT), Maa-Lana (ML) and Local Variety (LV) declined considerably from the 10th - 12th WAP following the onset of heavy rains, and increased slightly as the rains subsided. The increment of vertical growth at moderate rainfall and decline at excess rainfall, is in line with the report that Frafra potato crops perform well under moderate rainfall within 700-1,100 mm per annum (Enyiukwu *et al.*, 2014; Safwan and Mohammed, 2016).

3.11 Rainfall and vine branching in Frafra potato

In all the six varieties, vine branch development increased slowly from 4-6 WAP at varying degrees (Fig3.3). With the exception of NT variety, vine branching of other varieties increased sharply from the 6th WAP till the 10th WAP, when rains were in excess. Similar to their vertical growth, the MM and WP varieties from the 6th WAP increased steadily in vine branching until the 12 WAP (Fig 3.3). However, vine branching in the NT variety only increased appreciably from the 8th WAP to the peak at 12th WAP. The reduction in rainfall at the 8th WAP did not appreciably affect branching of vines in all six varieties (3.3).

Fig 2.3: Rainfall and vine production in Frafra potato

Compared to other varieties, the MM and WP produced more vine branches from the 8th – 10th WAP, supporting earlier report that these varieties are drought-resistant and require moderate rainfall conditions for growth and development (Enyiukwu *et al*., 2014; Safwan and Mohammed, 2016; Nyawudzo *et al*., 2019). There was a sharp decline in vine branching from the 12th WAP, and could be attributed to the initiation of reproductive growth which requires more assimilates for tuber formation and development, instead of vine production.

3.12 Rainfall and plant canopy spread in Frafra potato

The canopy spread of the six varieties were at steady rate from 4th to the 8th WAP (Fig 3.4). However, canopy development in four (4) of the varieties (ML, MM, WP and LV) were slow in the 8th WAP probably due to limited rainfall. However, the canopy of the NP variety continued to spread from 4th WAP until the 12th WP (Fig 3.4).

Fig 2.4: Rainfall and plant canopy spread in Frafra potato

The rate of growth of the canopy of the NT variety even increased at the 8th WAP despite the drought but rather decreased after the 10th WAP when heavy rainfall set in. Thus, with the exception of the MM variety, whose canopy growth rate increased after the heavy rainfall, the latter does not appear to promote canopy expansion in the rest of the varieties of Frafra potatoes

In this study, there were generally very massive vegetative development across the six Frafra potato varieties. Though Frafra potato fresh vegetative parts are said not to be a choice for livestock, such massive vegetative parts could be processed into animal feed (Enyiukwu *et al*., 2014).

3.13 Tuber production in frafra potato

The Fig 3.5 shows the mass of tubers produced by each of the six Frafra potato varieties. The LV variety produced the highest average mass of 28.80 Kg of tubers, whereas the lowest tuber mass of 19.15 Kg was recorded in the MM variety.

Fig 2.5: Tuber mass production in Frafra potato

Generally, there were massive vegetative biomass production of the varieties at the expense of tuber formation, probably attributable to the excessive rainfall at the reproductive phase of the crop, from the 10th WAP. The plants started flowering from 84 ±5 days in NT variety to 93 ± 3 days in NP variety after planting; and were ready for harvest at 138 ± 1 to 145±1 days after planting (Table 3.2).

This shows that though moisture is necessary for tuber development in Frafra potato, moderate moisture, particularly at the reproductive phase of crop promotes better tuber mass and size. This is in line with the report that tuber production in Frafra potatoes is highly dependent on the quantity and pattern of rainfall distribution, with moderate and evenly distributed rains favouring good tuber production, while heavy rainfall is counter-productive (Alleman, 2002; Nkamah, 2004; Enyiukwu et al., 2014).

Conclusion

The six Frafra potato varieties produced massive vegetative biomass and considerable yield of tubers under the rainfall condition. In this study the LV, NT and NP varieties performed much better in tuber formation under the rain-fed conditions, and are selective for farmers for rainy season production. Moderate rainfall, especially at the reproductive phase of the crop can promote better tuber mass and size, than excess moisture. The LV variety produced the highest tuber yield but recorded the lowest vegetative biomass. The MM and WP varieties produced the highest vegetative organs but the lowest tuber yield. The NT variety had moderate vegetative structures

and tuber yield. This result is an indication that if given the needed attention, the Frafra potato crop can produce high tuber yields to augment household food security, and provide fodder as well for livestock.

Recommendations

Base on the results, the following recommendations are worth considering:

i. The local variety (LV), Nachim –Tiir (NT) and Nutsuga Peisa (NP) varieties had better tuber yields and are selective for rainy season production

ii. Planting should be timely to escape excessive rains during the tuber initiation and formation stages of the crop

iii. Soil testing and nutrient management should be encouraged at farmer level to improve yields

Competing Interest

Authors have declared not having any competing interest.

Acknowledgments

We are grateful to the Savanna Agriculture Research Institute (SARI) for graciously providing us with five of the six frafra potato varieties studied. We are also thankful to the Department of Ecological Agriculture of Bolgatanga Technical University, for granting us the use of their experimental field. We acknowledge the following members of BTU Research Group 2015 who are not authors of this paper: Gabriel Asumboya, Abdul-Rahaman Saibu Salifu, Akazotiyele Richard and Atingah Abi Christina.

References

1. Alleman J, Hammes PS. Effect of photoperiod on tuberization in the Livingstone potato (Plectranthus esculentus N.E. Br). Field Crops Res. 2006;98:76-81.
2. Alleman J. Evaluation of Plectranthus esculentus N. B. Br as a potential vegetable crop. Ph.D. Thesis, University of Pretoria South Africa 2002;pp 40.
3. Ayimbire A, Asumboya G, Atinga CA, Akazotiyele R, Bodieu M. Growth and Yield Characteristics of Five Different Sweet Potato Varieties Grown in the Guinea Savanna Ecological Zone of Ghana. J Exp Agri Int. 2021;43(2):44-55. Article no. JEAI.67289 ISSN: 2457-0591.
4. Dumbuya G, Sarkodie-Addo J, Daramy MA, Jalloh M. Growth and yield response of sweet potato to different tillage methods and phosphorus fertilizer rates in Ghana. Journal of Exp Bio & Agr Sci. 2016;4(5):475-483.
5. Enyiukwu DN, Awurum AN, Nwaneri JA. Potentials of hausa potato (solenostemon rotundifolius (poir.) j. k. morton and management of its tuber rot in Nigeria. Green J Agron For & Hort 2014 Apr;2354-2306: 2(2):027-037.
6. Fery M, Choate J, Murphy E. A Guide to Collecting Soil Samples for Farms and Gardens. Oregon State University Extension Service. 2018 Oct:1-5.
7. Fleck S, Raspe S, Cter M, Schleppi P, Ukonmaanaho L, Greve M. Leaf area measurements: Part XVII. Thünen Institute for World Forestry, Germany. 2012:37.
8. Ghana Statistical Service (GSS). 2010 population and housing census (PHC). 2014.
9. Hua L, Hadziabdic Đ, Amissah N, Nowicki M, Boggess SL, Staton M, et al. Characterization of fifteen microsatellite loci and genetic diversity analysis for the Ghanaian food security crop Solenostemon rotundifolius (Frafra potato). Afr J Biotech. 2018;17(47):1352-1357. DOI: 10.5897/AJB2018.16666. http://www.academicjournals.org/AJB.

10. Isa M, Setiado H, Putri LA. The effect of internode number and the grow angle on the growth and yield of several sweet potatoes (Ipomoea batatas L.) varieties. J Agron. 2015;4:45- 52.
11. Kana HA, Aliyu IA, Chammang HB. Review on neglected and under-utilized root and tuber crops as food security in achieving the millennium development goals in Nigeria. J Agri Vet Sci. 2012;4:27-33.
12. Kwarteng AO, Ghunney TR, Amoah A, Nyadanu D, Abogoom J, Nyam KC, et al. Current knowledge and breeding avenues to improve upon Frafra potato (Solenostemon rotundifolius (Poir.) J. K. Morton). Gen Resour Crop Evol. 2018 Feb. Springer Science+Business Media B.V., part of Springer Nature. DOI 10.1007/s10722-017-0581-6.
13. Motsara MR, Roy RN. Guide to laboratory establishment for plant nutrient analysis. Food and Agriculture Organization of the United Nations Rome, Italy. Fertilizer and Plant Nutrition Bulletin 19. 2008.
14. Nkansah GO. Solenostemon rotundifolius (Poir), PROTA 2: Vegetables/legumes (CD-ROM). PROTA, Wageningen, Netherland. 2004.
15. Nyawudzo G, Amissah JN, Kusi F, Ofori1 K. Morphological and molecular characterization of frafra potato (Solenostemon rotundifolius) accessions in Ghana. Sci & Dev't. 2019:3.
16. Ochieng LA. Agro-morphological characterization of sweet potato genotypes grown in different ecological zones in Kenya. J Hort & Plant Res. 2019;5:1-12. ISSN: 2624-814X, DOI:10.18052/www.scipress.com/JHPR.5. 1
17. Safwan II, Mohammed UA. Review on the nutritional value, cultivation and utilization potential of some minor and under-utilized indigenous root and tuber crops in Nigeria. Int J Adv Res. 2016;4(3):1298-1303. http://www.journalijar.com

18. Su W, Liu Y, Lei J, Wang L, Chai S, Jiao C. et al. Phenotypic variation analysis of sweet potato germplasm resources from different agro-climate zones in the world. Am J Exp Agri. 2016;13(6):1-13.
19. Sugri I, Kusi F, Kanton RAL, Nutsugah SK, Zakaria M. Sustaining frafra potato (solenostemon rotundifolius poir.) in the food chain; current opportunities in Ghana. 2013: The 3rd international conference on neglected and underutilized species (nus): for a food-secure Africa: 2013 Sept; Mensvic Grand Hotel, Accra, Ghana. 2013. P. 25 – 27.
20. Sugri I, Kusi F, Kanton RAL, Nutsugah SK, Zakaria M. Sustaining frafra potato (Solenostemon rotundifolius poir.) in the food chain; current opportunities in Ghana. J P Sci. 2013 Sept;1(4): 68-75. doi: 10.11648/j.jps.20130104.14.
21. Taylor and Francis Group. 2nd Edition. Carter MR, Gregorich EG, editors. Soil sampling and methods of analysis canadian society of soil science. 2008. https://www.cambridge.org>article.
22. Tindall HD. Vegetables in the Tropics. London, UK: Macmillan Press Limited. 1983; 242–245.
23. Tortoea C, Akonora PT, Kusib F, Anabirec PA, Owusub RK, Boatenga C. Unearthing the potential of the frafra potato (Solenostemon rotundifolius) flour in culinary application: sensory and nutritional analysis of its pastry products. J Cul Sci & Tech. 2020;18(1):1–12. https://doi.org/10.1080/15428052.2018.1495588.
24. Widaryanto E, Saitama A. Analysis of plant growth of ten varieties of sweet potato (Ipomoea batatas L.) cultivated in rainy season. Asian J Plant Sci. 2017;16:193-199.

Figure Click here to access/download;Figure;S1 Figures.docx

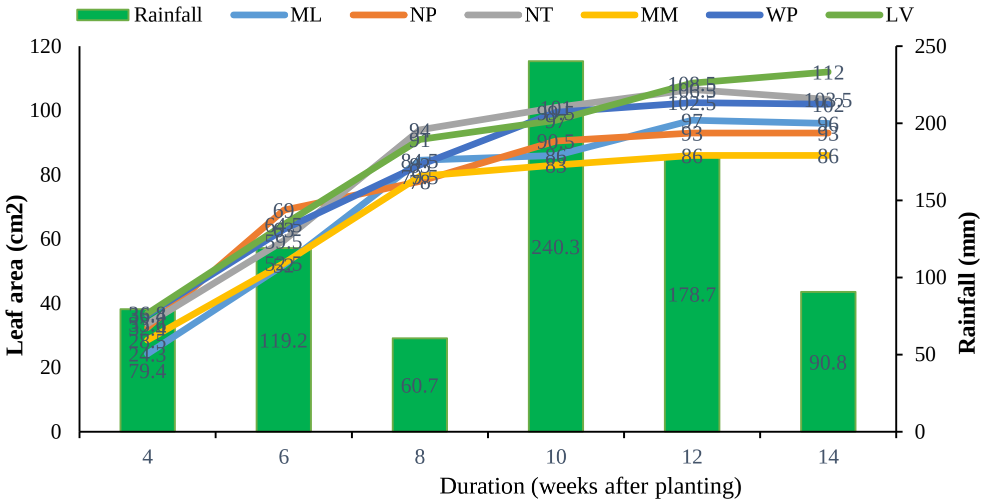

S1 Figure. Fig 2.1: Rainfall and leaf area expansion in Frafra potato

LV = Local variety, ML = Maa-Lana, MM = Manga-Moya, NP = Nutsuga Peisa, NT = Nachim – Tiir, WP = WAAP Peisa

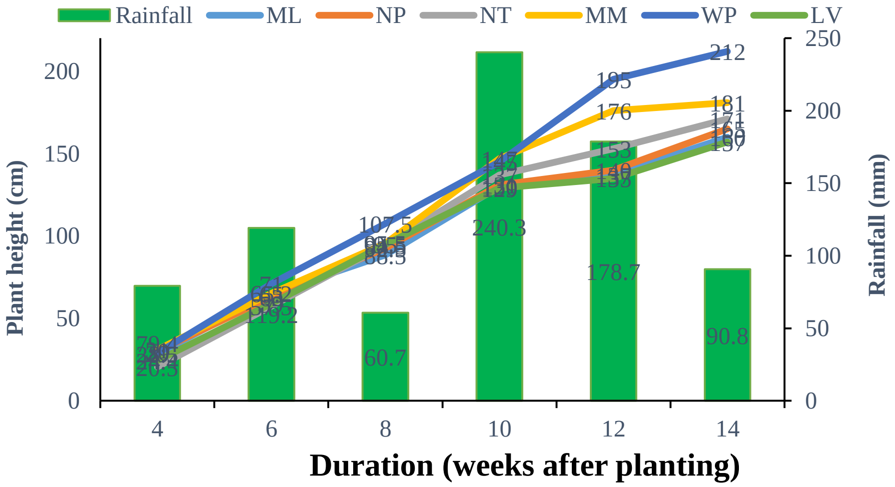

S1 Figure. Fig 2. 2: Rainfall and plant height in Frafra potato

LV = Local variety, ML = Maa-Lana, MM = Manga-Moya, NP = Nutsuga Peisa, NT = Nachim – Tiir, WP = WAAP Peisa

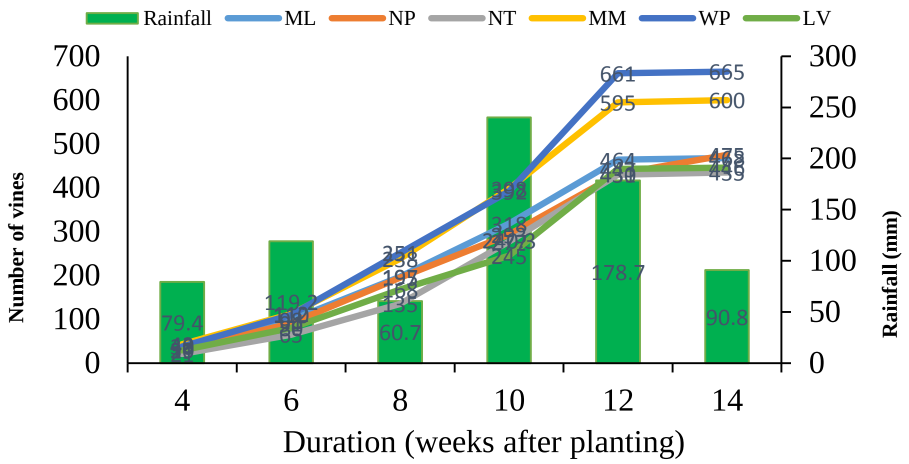

S1 Figure. Fig 2.3: Rainfall and vine production in Frafra potato

LV = Local variety, ML = Maa-Lana, MM = Manga-Moya, NP = Nutsuga Peisa, NT = Nachim – Tiir, WP = WAAP Peisa

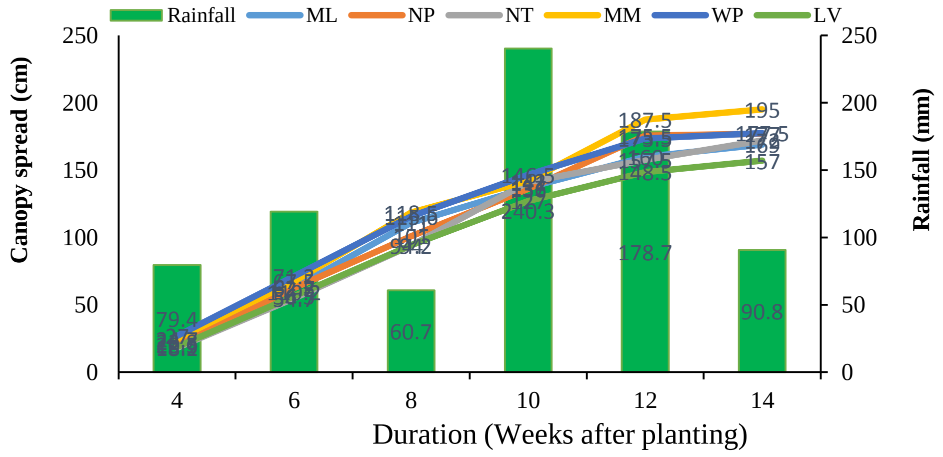

S1 Figure. Fig 2.4: Rainfall and plant canopy spread in Frafra potato

LV = Local variety, ML = Maa-Lana, MM = Manga-Moya, NP = Nutsuga Peisa, NT = Nachim – Tiir, WP = WAAP Peisa

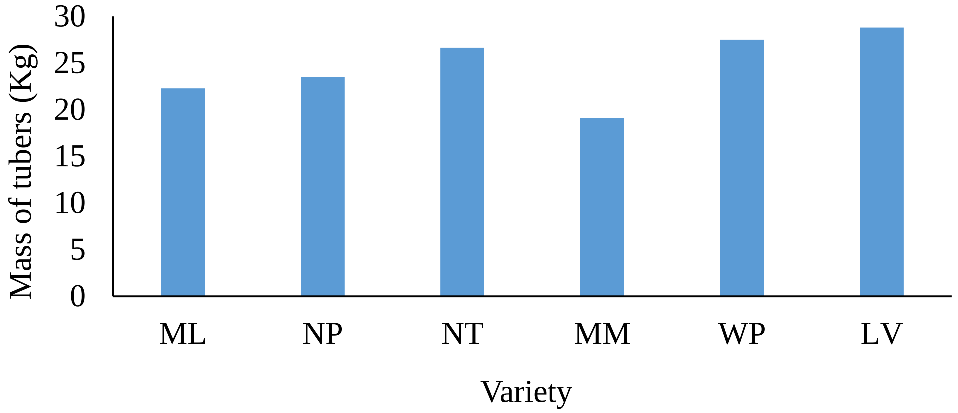

S1 Figure. Fig 2.5: Tuber mass production in Frafra potato

LV = Local variety, ML = Maa-Lana, MM = Manga-Moya, NP = Nutsuga Peisa, NT = Nachim –Tiir, WP = WAAP Peisa

Figure

Click here to access/download;Figure;Supporting information.docx

Supporting information

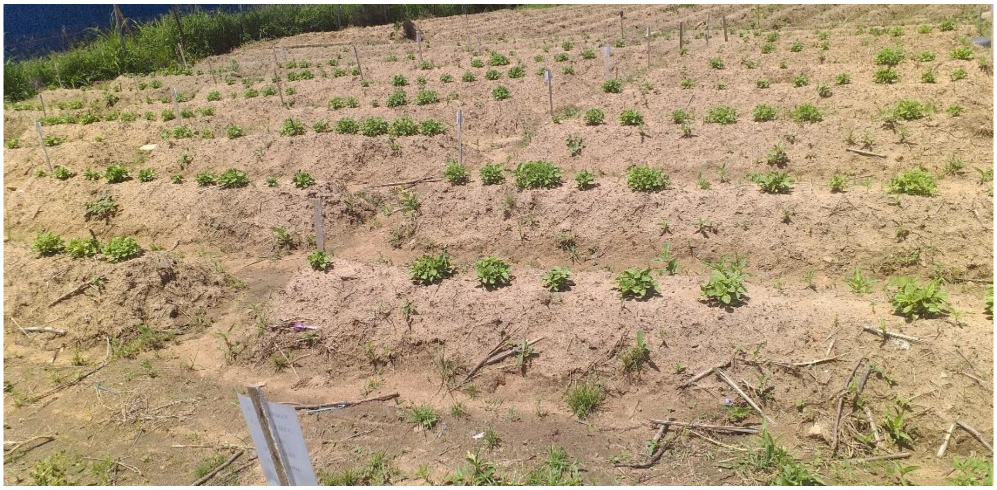

Plate 1: The Frafra potatoes five weeks after planting

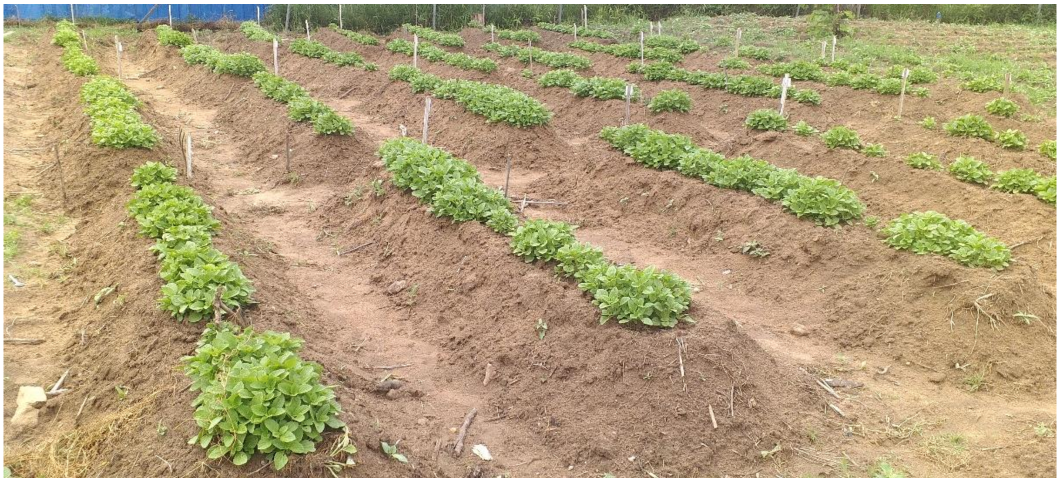

Plate 2: The Frafra potatoes seven weeks after planting

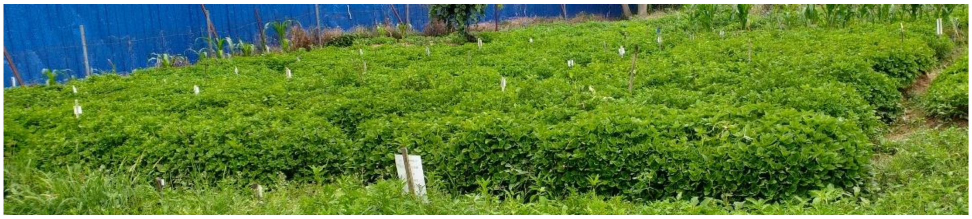

Plate 3: The Frafra potatoes three months after planting

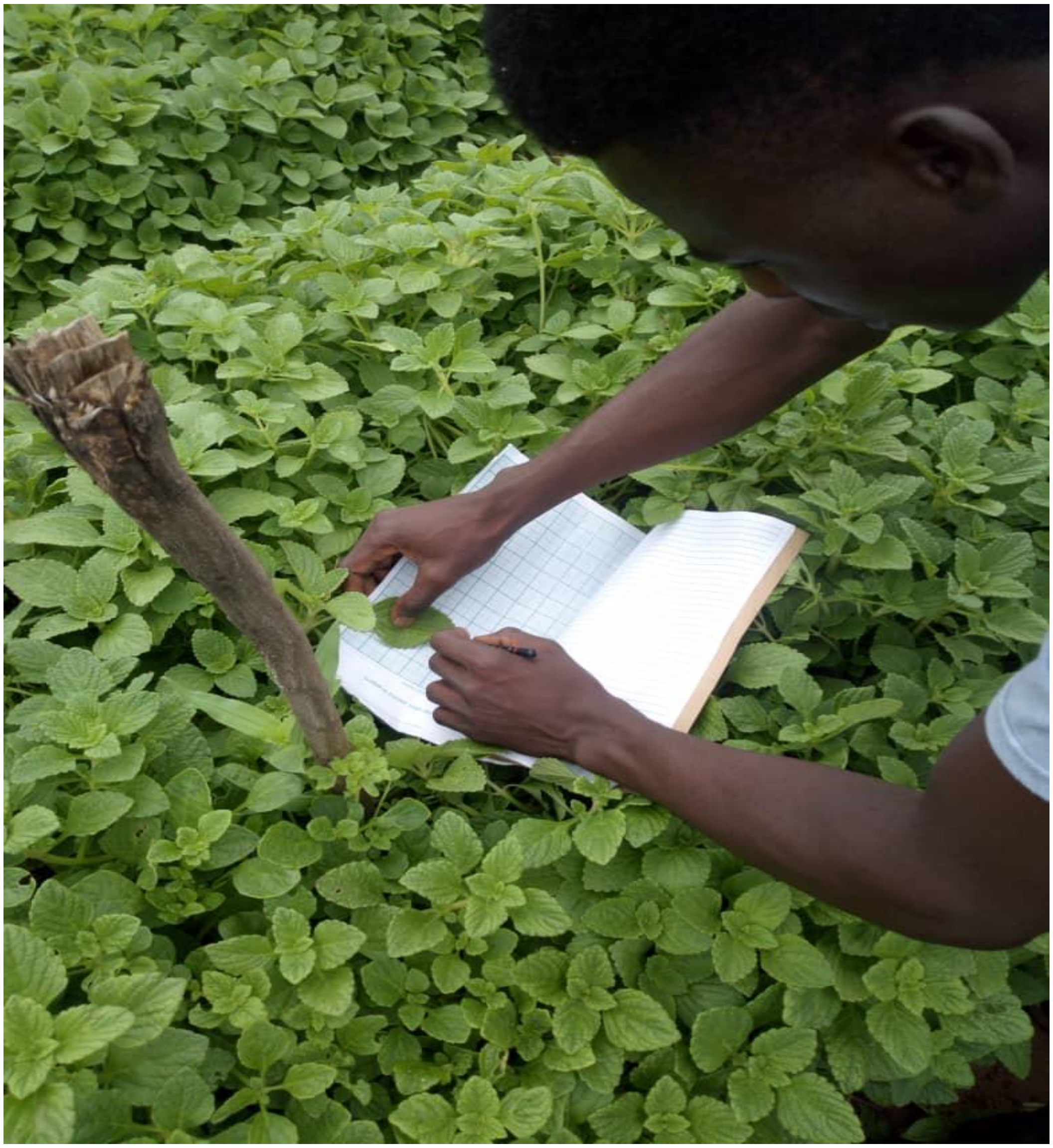

Plate 4: Tracing the outline of a leaf on a graph in order to estimate its area.

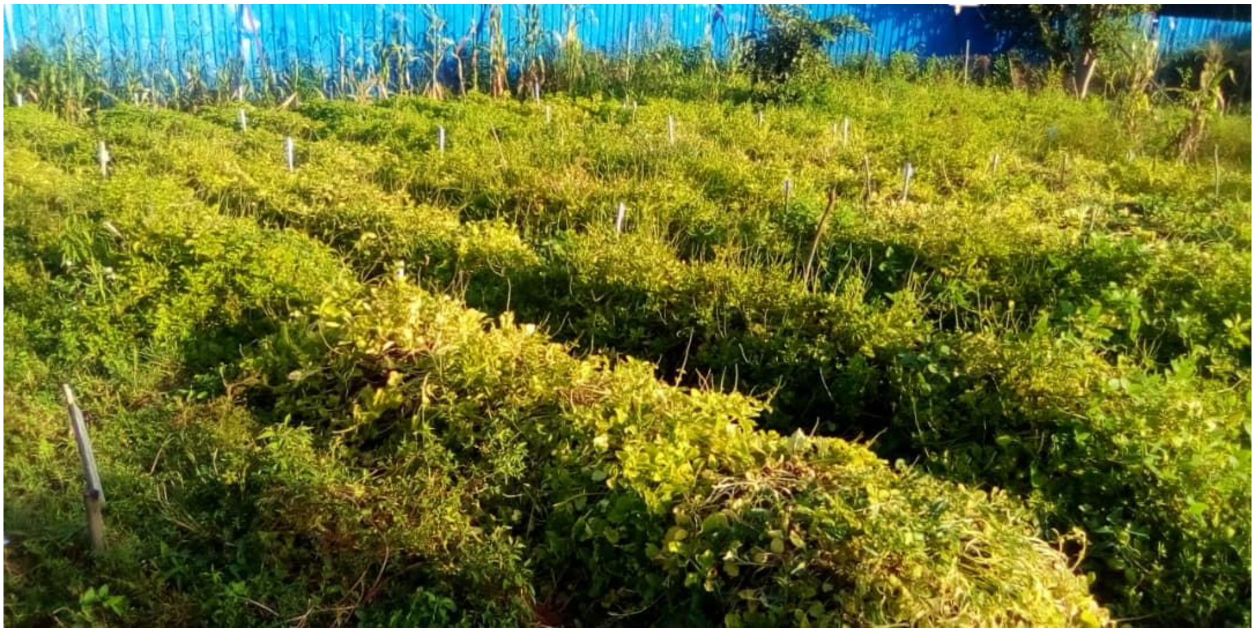

Plate 5: The Frafra potatoes at maturity

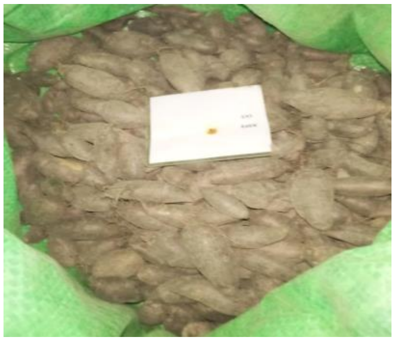
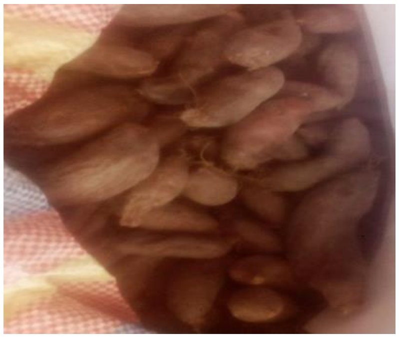
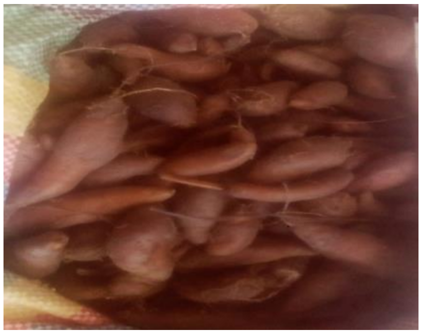

LV ML MM

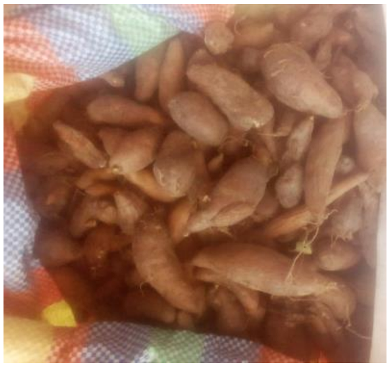
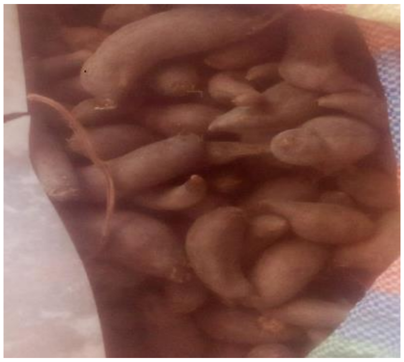
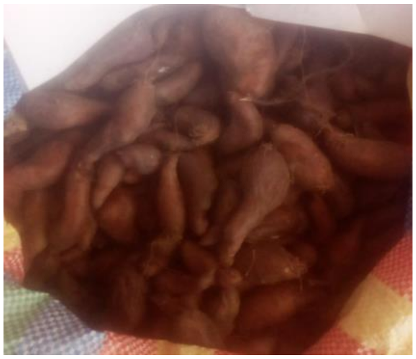

NP NT **WP**

Plate 6: Tubers of the studied six varieties of Frafra potato.

LV = Local variety, ML = Maa-Lana, MM = Manga-Moya, NP = Nutsuga Peisa, NT = Nachim –Tiir, WP = WAAP Peisa
