# Supplementary material for: Comparing the growth and yield performance of six different varieties of frafra potato (Solenostemon rotundifluis Poir) grown under rain-fed conditions in the Guinea Savanna ecological zone of Ghana
Source: PLoS One. 2022 Nov 16;17(11):e0276566. doi: 10.1371/journal.pone.0276566 (PMC9668201; doi:10.1371/journal.pone.0276566)
Supplement: Attachment — Submitted filename: RESPONSES TO REVIEWERS.docx [file pone.0276566.s008.docx]

**RESPONSES’ TO REVIEWERS COMMENTS**

|  | To take advantage of our partnership with Editage, visit the Editage website ([www.editage.com](http://www.editage.com)) | Manuscript has been edited by Editage. Therefore, I present two copies as follows:   i. Revised manuscript with Track Changes. This was corrected by the authors to effect the reviewers’ comments. ii. Manuscript. This manuscript is an unmarked copy (without track changes). This is the revised, editage edited copy which is submitted now to be considered for publication. |
| --- | --- | --- |
| **No.** | **Comment of reviewer one** | **Author’s response** |
| 1 | Too many Kew words | Thanks. Please, they have been reduced to seven |
| 2 | 2 must be supperscribed | Thanks. Please, it has been done (cm^2^) |
| 3 | Gramatically not correct. b/s single variety require singular verb was. Or the sentence has to be rewritten. | Thanks very much, please. However, the statement “Plants of WAAP Peisa were tallest (31.7 cm) as those of Local Variety were shortest (25.0 cm)”, is Grammatically correct because the verb “were” refers to the noun “plants” of WAAP Peisa, in the first part of the sentence which is replaced by the pronoun “those” in the second part of the sentence. Therefore, the use of verb “were” in both parts of the sentence is correct. |
| 4 | 'with' must be changed to 'while' | Thanks. Please, I have done so |
| 5 | you must reason out in the result or duscaution why? leaf area and yield is negetively related b/s it must be + related | Thanks. Please, this has been well explained in the results and discussion section. |
| 6 | why? u are measuring the area of the leaf. area means the total coverege of the leaves and u selected one leaf for one replication which is not reponsible leaf nuber to detemine leaf area. B/s a plant has leaf size difference from bottom to top. so that for one plant it need at least three leaf top matured leaf, meduim and botom leaves samplimg. In my opinion the result obtaioned from this sumpling is not have value. | Thanks. Please, I agree that using three leaves may cover the various sizes of the leaves at the three stages of growth. However, using one leaf also gives a good estimate because at the begining of measurement, the leaf was young and small and its size was measured. Then, that same leaf grew to maturity and because the measurement was periodical, the various sizes of the leaf at the different stages of growth were measured. Again, each variety had three replicates and a leaf was measured from each replicate which means three leaves were measured from each variety. Given that the comparison was between varieties, the average area of three leaves was actually used, which is in line with your opinion. |
| 7 | you should site the tables with results respectively. You should have to have also the results wihich show the significance level in your table following all the parametters you measured and analyized. | Thanks. Please, these have been done. |
| 8 | Why one-quarter of 1 cm^2^ was excluded in the estimation of leaf area? | Thanks. Please, in the estimation, where the leaf outline covered only three-quarters of the 1 cm^2^, they were considered as fully covered. This means one-quarter of each of these squares was included in the leaf area even though they were outside the leaf outline. Therefore, where only one-quarter of the 1 cm^2^ was covered by the leaf outline, this was excluded to balance the other one-quarter of 1 cm^2^ that was included though it was outside the leaf outline. |
|  |  |  |
|  | **Comment of reviewer two** | **Author’s response** |
| 1 | Proof reading is highly recommended to minimize the grammar and punctuation errors/weakness, which is generally conspicuous in write up | Thanks. Please, proof-reading will be done. |
| 2 | Line 29: the sentence may end at: deprived soils | Thanks. Please, this has been done, |
| 3 | Line 34&38: Punctuate: , respectively | Thanks. Please, I have done so. |
| 4 | Line 46: I suggest the last sentence should be replaced by the sentence at Line 522-524. But should exclude the part that read ‘’and provide fodder as well for livestock”. In fact, animals hardly feed on the leaves of Frafra potato, due to the off-flavour | Please, I have done so.  However, the issue of whether animals eat Frafra potato leaves or not, is debatable. Animals, when they have choices to make, will reject some foods. However, in places where the extensive or semi-extensive systems of rearing animals is practiced, and where the dry season covers 7 or 8 months, at the peak of the dry season, animals eat any plant matter they come across. Therefore, if they are served with Frafra potato leaves, they eat voraciously. |
| 5 | Line 72: animal feed? | Please, I have explained this above. In the dry season, animals in my area eat Frafra potato leaves voraciously. |
| 6 | Line: 74-75. The information may not be correct. (sentence can modified or deleted) : the tubers rot if not harvested early under the hot temperature in northern Ghana | Thanks. Please, this is a finding by Sugri et al. (2013). They found out that some farmers did so. Living in northern Ghana myself, I can confirm that when the rains stop in the dry season, there is no adequate moisture in the soil to sustain microbial activities to cause rotting! What I have experienced in my area is that the tubers lose water and shrink in sizes. The soil dries up and locks the tubers in the soil. Therefore, depending on the nature of the soil, you may find it difficult to dig and harvest the tubers if they are left in the field for a long time in the dry season. However, if it rains in the dry season, then the tubers immediately start rotting. |
|  | Line 79: These qualities | Thanks. Please, I have corrected it |
|  | Line 124: average night temperature of 14 ^o^C. Please check again | Thanks. Please, this is from a Statistical Service document published in October 2014 on the 2010 population census. I do appreciate your concern as northern Ghana is known for her high temperatures. However, during harmattan, night temperature can be even lower than 10 ^o^C. However, due to global warming, such chilly weather is now rare. |
|  | Figures.2.1 to 2.4: Titles should read: Relationship between rain fall and leaf area expansion , etc | Thanks. Please, this has been revised to read: Relationship between rainfall and leaf area expansion in Frafra potato, etc. |
|  | Fig. 5.2 Tuber mass. Not clear to me. I do not understand what this is about. | Thanks. This has been revised to read: Fig. 2.5: Mass of tuber produced by the six varieties of Frafra potato |
|  | **Other comments** | **Author’s responses** |
|  | Abonuusum et al., 2021 not presented in reference list | Thanks. Please, I am sorry for using the wrong name, Abonuusum instead of Ayimbire, which listed as number 3 in the references |
|  | Line 275 (now 262) cite plates 1, 2, 3, … | Thanks. Please, this has been done. |
|  | Line 325 following: cite tables with the results and add a table showing significant differences. | Thanks for drawing my attention to the need to cite the table of results and add P-Values. These have been done, I have added a row of P-Values at the bottom of table 3.2. Hope it meets your request. |
|  | CV= 56% …. CV measures experimental error and not variation…. | Thanks. Please, this has been done. Have change the sentence and used P- Value instead. |
|  | Line 415 following: May be nutrient imbalance. Excessiveness in nitrogen…. | Thanks very much for drawing my attention to the possibility of excessive nitrogen being the cause of the massive vegetative growth at the expense of tuber formation. However, table 3.1 clearly shows that the soil was poor in nutrient content. No chemical fertilizer was also applied. Therefore, excessive nutrient in the soil could not have been the cause of the high vegetative growth. |
|  | Line 425: Remove Plate and number 6 | Please, the statement: fig 2.1: Rainfall and leaf expansion in Frafra potato, is not a subheading. It indicates where fig 2.1 should be placed. Currently, fig 2.1 is attached as additional information after the references. Therefore, it is not clear to me when you said I should removefig 2.1, and leave the sentence hanging like that. Thank you |
|  | Nkamah, 2004 is not in reference list | This is cited in the list of references (number 14). Thank you |
|  | Lines 550 -553 Ayimbire A, Asumboya…. Not cited in text | Thanks. Please, it was wrongly cited in text as Abonuusum et al., 2021, now corrected. |
|  | We note that Figure 1 in your submission contain [map/satellite] images which may be copyrighted | Please, figure 1 has been replaced with a map obtained from the Ghana Statistical Service, Upper East Region. Thank you |
|  | We note that Supplementary Figure plate 4 includes an image of a participant in the study……… The signed consent form should **not** be submitted with the manuscript, ….. Please amend the methods section and ethics statement of the manuscript to explicitly state that the patient/participant has provided consent for publication | Mr. Abdul Ganiu Anyagri Ndeogo has given written informed consent (as outlined in PLOS consent form) to publish his image in Supplementary Figure plate 4. The ethics statement has been amended. Thank you |
|  | Please upload a copy of Figure 3, to which you refer in your text on page 22 | Please, it is figure 2 that was mistakenly cited as figure 3. Thank you |
|  | Please ensure that you refer to Figure 2 in your text | Please, figure 2 was mistakenly cited as figure 3. This has been corrected. Thank you |
|  | One of the noted authors is a group or consortium BTU Research Group 2015. In addition to naming the author group, please list the individual authors and affiliations within this group in the acknowledgments section of your manuscript. | Please, BTU Research Group 2015, is not an author of the manuscript. It is a research group in the university some of whose members are authors of this manuscript. The other members are **not** authors of this manuscript. Thank you. |
